# Addressing medical absenteeism in pre-vocational secondary students: effectiveness of a public health intervention, using a quasi-experimental design

**DOI:** 10.1186/s12889-016-3718-1

**Published:** 2016-10-21

**Authors:** Yvonne T. M. Vanneste, Jolanda J. P. Mathijssen, Ien A. M. van de Goor, Carin M. C. Rots – de Vries, Frans J. M. Feron

**Affiliations:** 1 Regional Public Health Service West Brabant, PO Box 3024, 5003 DA Tilburg, The Netherlands; 2 Faculty of Social and Behavioural Sciences, Tranzo, Tilburg University, PO Box 90153, 5000 LE Tilburg, The Netherlands; 3 Faculty of Health, Medicine and Life Sciences (FHML), CAPHRI, Department of Social Medicine, Maastricht University, PO Box 616, 6200 MD Maastricht, The Netherlands

**Keywords:** School absenteeism, Medical absenteeism, Preventive youth health care, Intervention research, Health inequalities, Pre-vocational secondary education

## Abstract

**Background:**

Students’ health and school absenteeism affect educational level, with adverse effects on their future health. This interdependence is reflected in medical absenteeism. In the Netherlands, a public health intervention has been developed to address medical absenteeism in pre-vocational secondary education. This study aims to investigate the effectiveness of this intervention on students’ medical absenteeism, compared to “medical absenteeism policy as usual”.

**Methods:**

A quasi-experimental design with an intervention group (493 students) and a control group (445 students) was applied. Multilevel analysis was used to study differences in the development of the level of a student’s medical absence over time (after 3 and 12 months).

**Results:**

In the intervention group, the level of absenteeism decreased from 8.5 days reported sick in 12 school weeks to 5.7 days after 3 months, and to 4.9 days after 12 months. The number of absence periods fell from 3.9 in 12 school weeks to 2.5 after 3 months, and to 2.2 after 12 months. In the control group, the absence days initially decreased from 9.9 days reported sick in 12 school weeks to 8.4 days after 3 months, after which an increase to 8.9 days was measured. The number of absence periods initially decreased from 4.5 in 12 school weeks to 3.5, after which an increase to 3.7 was measured. The number of absence days per period remained about the same in both groups.

**Conclusions:**

The study provides first indications for the intervention to be effective for Dutch pre-vocational secondary students with increased medical absence rates. The intervention, which consists of personalised management of medical absenteeism by systematic identification of students with extensive medical absenteeism and consistent referral to youth health care physicians, appears to reduce the absence rates more effectively than “medical absenteeism policy as usual”. The effectiveness of the intervention is shown primarily by a decrease in the number of periods reported sick.

## Background

School absenteeism may lead to a lower level of education or even school dropout [1–4]. Low educational level and school dropout are both strongly associated with an increased risk of social failure and delinquency [5–9], increased risk behaviour (e.g. smoking, obesity, lack of exercise) [10, 11], a higher prevalence of mental problems and chronic health issues [12–15], and higher mortality rates [16–21]. Tackling school absenteeism can reduce socio-economic health inequalities by optimising children’s educational opportunities and future prospects.

Literature frequently distinguishes between unexcused and excused school absenteeism. Unexcused absenteeism is truancy, not attending school without permission. Excused absence means not attending lessons with permission because of familial or personal reasons, with sickness reporting, also called medical absenteeism, as a major reason. Prevalence rates on school absenteeism as reported in literature are not directly comparable internationally, due to inconsistent use of parameters for school absenteeism. Nowadays, figures at the national level show that medical absenteeism occurs approximately twice as often as truancy [22–24]. In the Netherlands in 2002 medical absenteeism was more prevalent in pre-vocational secondary education than in the other two tracks in secondary education (senior general secondary education and pre-university education): 4 v. 3.7 and 3.4 % [25]. See Appendix for a description of the Dutch educational system [26]. No more recent valid figures on absenteeism are available. Because of the strict enforcement of measures against truancy in recent years and on the basis of current international figures, it is conceivable that in the Netherlands also, the medical absence rate has increased meanwhile compared to truancy.

Research on medical absenteeism shows that it is caused not only by medical problems [2, 27], but also by a low threshold for reporting sick, by risk behaviour, or stress-related physical complaints [2, 22]. The latter may have more complex causes such as psychological, family, or social problems [2]. Students attending Dutch pre-vocational secondary education are less positive about their health, have a poorer lifestyle and more psychosomatic complaints [28, 29], and more often demonstrate behavioural problems [30], which may explain their higher medical absenteeism. Moreover, these students have difficulties planning their studies [31]. Consequently, they experience problems in catching up after once having fallen behind because of missing course material. When dropping out, these students cannot fall back on a lower level of education. This explains why dropout rates are the highest in pre-vocational secondary education. On average three quarters of the new early school-leavers that are registered each year come from lower pre-vocational secondary education [32].

Until now, the only programmes for reducing school absenteeism developed and applied relate to unexcused absence and school refusal behaviour [2]. It was recommendable to develop interventions for addressing medical absenteeism, especially for pre-vocational secondary students, because of its increased prevalence and risk of dropout. In the Netherlands, medical absenteeism from secondary school depends solely on parental sick reporting and the authority to decide how to deal with it lies with the schools. This so-called “medical absenteeism policy as usual” is not defined. Dutch secondary schools have their own care system and their own way of handling sick reports. The Youth Health Care department of the Regional Public Health Service West Brabant (a region in the southwest of the Netherlands with a population density of 375 people per square kilometre) developed the MASS intervention [33] (see Table 1) in collaboration with the educational sector. It was assumed student’s absence rate to be reduced by providing personalised care, support, and guidance addressing these multiple causes. By applying the MASS intervention, students with increased medical absence rates (i.e. four periods in 12 school weeks, or more than six consecutive days) are identified by the school, and are then referred to youth health care physicians (YHCPs). Besides initial medical assessments, the YHCP also perform biopsychological evaluation of the absence in order to identify specific needs of the student. In addition, the physician, together with student, parents and school, designs a management plan based on guidelines for care, counseling, and educational adaptation. This plan describes what is needed to improve the student’s health and well-being and to maximise the participation in school activities, thus protecting the student against underachievement and dropout from education. Recently, Hawkrigg and Payne presented a more or less similar practical approach to prolonged school non-attendance [34]. The MASS-criterion “four periods in 12 school weeks” was chosen because practice shows that the study outcomes of students are adversely affected not only by the length, but also by the frequency of (short-term) absences, therefore both presenting similar risks. The MASS-criterion “more than six consecutive days” was chosen to exclude students with uncomplicated influenza illness, because uncomplicated influenza illness typically resolves after 3–7 days for the majority of persons [35]. The MASS intervention was included in a survey on European real-life interventions that aim to reduce socio-economic health inequalities [36].

**Table 1:** Description of the Dutch intervention ‘Medical Advice for Sick-reported Students’, abbreviated as MASS

The MASS intervention consists of an integrated approach in a public health setting. MASS provides a clear framework in which schools, in direct collaboration with youth health care physicians (YHCPs), are able to reach students and their parents, discuss aspects of the student’s medical absence, and design and monitor a management plan that aims to optimise students’ health and maximise students’ participation in school activities. In summary, the aim of the MASS intervention is to limit the absenteeism by arranging appropriate care, educational adjustments and adequate support for students and parents. A systematic routine is followed.
Step 1 School’s policy:
The school communicates with students and parents about the new policy in case of absenteeism because of medical reasons.
Step 2 Referral to the YHCP:
Students with extensive medical absence are identified by school by using well-defined threshold criteria: reported sick four times in 12 school weeks or more than six consecutive school days (MASS-criteria). Meeting the criteria always leads to a referral to the YHCP for student and parents.
Step 3 Consultation of student and parents with the YHCP:
During the interview and medical assessment YHCPs look for biological, psychological and social factors that contribute to the students’ medical absenteeism. The YHCP identifies whether there is a specific somatic or psychiatric diagnosis to account for the absence. If the diagnosis is clear the focus will be on optimising the (adherence to) treatment. In cases of frequent physical complaints and psychosocial problems with no clear medical diagnosis, the YHCP considers diagnostics, and looks for family and school related factors, as well as health risk behaviours and lifestyle aspects that contribute to the physical complaints and psychosocial problems. If needed, the YHCP refers to a medical specialist or a psychosocial support network. A management plan is then designed together with student, parents and school, and with curative professionals, if applicable. This plan includes agreements on cure, care and school attendance.
Step 4 Monitoring the management plan:
School and YHCP monitor the execution of the management plan.

To the best of our knowledge, the effectiveness of an intervention for addressing extensive medical absenteeism has not been investigated before. The objective of the current study is to evaluate the effects of the MASS intervention in pre-vocational secondary education: is there a decline in students’ level of medical absenteeism following systematic identification and consistent referral to the YHCP of students with extensive medical absenteeism, compared to a situation in which this specific approach is absent?

## Methods

### Setting

The study was conducted in the Netherlands, West Brabant region. In school year 2011–2012, the MASS intervention had been applied by seven out of all 21 schools for pre-vocational secondary education. To implement the intervention, schools had to release their own financial resources to pay for the consultation with the YHCPs. All these seven schools were asked to participate in the study as an intervention school. In 2014, within the group of the 14 remaining schools, seven schools were asked to participate in the study as a control school, by providing anonymised absence data of their students retrospectively. This was done to avoid influence of their ‘medical absenteeism policy as usual’ during the study period. A condition for participation in the study as a control school was that the MASS intervention still had not been applied in the school year 2012–2013. The selection of the control schools was determined by school characteristics, that had to match as much as possible with those of the intervention schools. The characteristics of the schools are described in Table 2.

**Table 2:** The characteristics of the intervention and control schools

School		School characteristics		
		Urbanisation^a^	Fields of education	Size of the school^b^
Intervention schools	School 1	predominantly rural region	General lower secondary education Social care	medium-sized
	School 2	predominantly urban region	General lower secondary education Economics Technology	medium-sized
	School 3	predominantly urban region	Economics Social care Technology	medium-sized
	School 4	predominantly urban region	General lower secondary education Economics Technology	large
	School 5	urban region	General lower secondary education Agriculture	medium-sized
	School 6	urban region	General lower secondary education	medium-sized
	School 7	urban region	General lower secondary education Economics	large
Control schools	School 8	urban region	General lower secondary education Sports	medium-sized
	School 9	predominantly urban region	General lower secondary education Economics Technology	large
	School 10	predominantly rural region	General lower secondary education Social care Technology	medium-sized
	School 11	predominantly rural region	General lower secondary education Agriculture	large
	School 12	predominantly urban region	General lower secondary education Agriculture	medium-sized
	School 13	predominantly urban region	General lower secondary education Economics Social care Technology	small
	School 14	rural region	Economics Social care Technology	medium-sized

^a^Urbanisation: rural (<200 inhabitants per square kilometre), predominantly rural region (200–500 inhabitants per square kilometre), predominantly urban region (500–1000 inhabitants per square kilometre) and urban (>1000 inhabitants per square kilometre)

^b^Size of the school: small (<250 students), medium-sized (250–750 students) and large (>750 students)

For both groups a prerequisite for participation in the study was a digitised and highly qualified absence registration system. All seven intervention schools and seven control schools eventually participated.

### Study design and population

Due to the active role of schools in the intervention, as explained above, random assignment of schools to “intervention or control schools” was not an option. Therefore the study had a quasi-experimental design. The intervention group consisted of students who were attending one of the intervention schools, had been identified by the school as having extensive medical absence according to the MASS-criteria, and had been referred to the YHCP during school year 2011–2012. The control group consisted of students who were attending one of the control schools, and who were identified retrospectively in the absence data as having extensive medical absence according to the MASS criteria.

### Data collection procedure and outcome measurements

In order to determine the level of medical absence, the school absence registration over the school years 2011–2012 and 2012–2013 was used. All school absence registration systems work by teachers who check per lesson and digitally record the students being present or absent. Partial absences such as going home during the day because of sickness were counted as full-day absences. When still reported sick after autumn, spring, and Christmas breaks, absences counted for new sickness reporting. Due to the shifting of summer holidays, the irregular school hours during the first school week and the examination weeks at the end of the school year, the level of medical absence was calculated only between September 1 and June 9. When the measurement period was (partly) outside this period, the absence rate was recorded as “missing”.

The level of a student’s medical absence was measured as the number of absence periods and absence days reporting sick during the 12 school weeks prior to the three measurement points. To ensure the accuracy of the measurements, to maintain the class conditions for the student the same as much as possible, and to reduce missings (for example as a result of the student moving to another school), a measurement period of 12 school weeks was chosen so that the effects at 12 school weeks’ time could consequently be measured as much as possible in the same school year. For the intervention group, the first measurement point (T_0_) was the consultation moment with the YHCP. At the end of the consultation written informed consent was obtained from students and parents to participate in the study. For the control group, the first measurement point (T_0_) was at two pre-defined points in time: December 1 or March 9. These points in time were chosen to avoid missing data in the 12 school weeks’ measurements periods. The data of a student with an extensive absence rate at both points in time were included in December 1. After 3 months (T_1_) and 12 months (T_2_) the measurement was repeated. In accordance with the Dutch public health act [37], collection of data to perform anonymous group comparisons does not require prior informed consent.

### Statistical analysis

Pearson’s chi-squared tests (for categorical variables) and Student’s *t*-test (for continuous variables) were used to determine differences in socio-demographic variables between the intervention group and the control group.

Due to the hierarchical structure of the data, with the repeated measurements on medical absenteeism (first level) nested within students (second level) nested within schools (third level), multilevel analysis (linear mixed models in SPSS, version 19.0) [38] was applied for studying differences between the intervention group and the control group in the development of the level of medical absence over time. The intra class correlations [39] were calculated at school and student level. Intervention, time and the interaction between intervention and time were added as fixed factors to the model. Schools and students were defined as random effects.

## Results

The intervention group consisted of 493 students (12 % of the total school population of 4159 students) and the control group consisted of 445 students (14 % of the total school population 3153 students). The total study group, including both intervention and control groups, consisted of 938 students, of whom 40 % were male and 60 % female. As shown in Table 3, there is a statistically significant difference between the two groups in gender, age, absence rate in periods, and absence rate in days. The intervention group had a lower proportion of females, a somewhat higher average age, and a lower absence rate in terms of both periods and days (initial values). There was no significant difference in the average number of days per period between the groups.

**Table 3:** The selected demographic characteristics and the initial values of medical absenteeism of the intervention and control groups

	Intervention group (493)	Control group (445)	Statistical values
Gender, % female	55.8*	64.3	*χ* ^2^ = 7.01**, df = 1
Age in years, mean (SD)	14.54 (1.32)	14.32 (1.28)	*t* = 2.49*
Absence rate in periods per 12 school weeks, mean (SD)	3.91 (1.62)**	4.50 (1.16)	*t* = −6.20**
Absence rate in days per 12 school weeks, mean (SD)	8.40 (5.39)*	9.92 (5.39)	*t* = −4.27**
Number of days per period	2.29 (1.53)	2.39 (1.67)	*t* = −0.95

* *p* ≤ 0.05; ** *p* ≤ 0.01

**Fig. 1:**
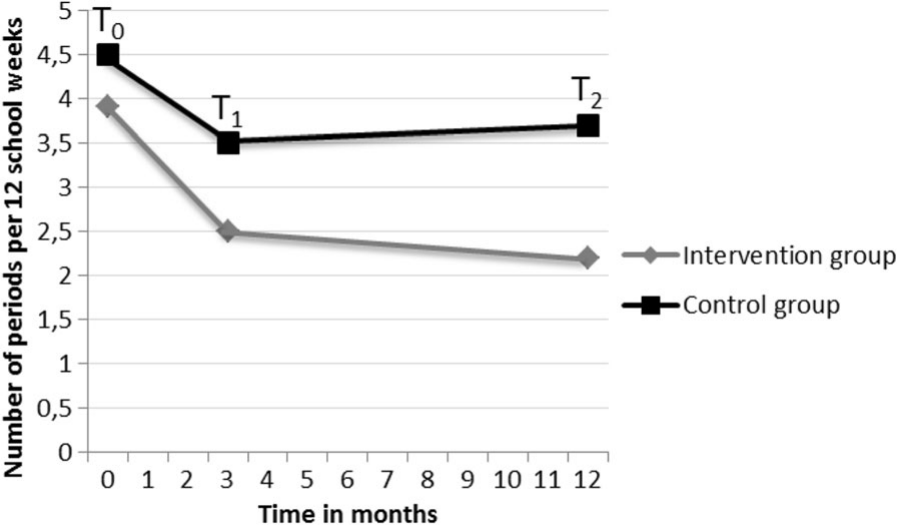
The progress of the absenteeism in number of periods

In the intervention group, 170 students (35 %) had been referred to the YHCP before December 1, 226 students (46 %) between December 1 and March 9, and 97 students (20 %) after March 9. In the control group, 335 students (75 %) were included on December 1, and 110 students (25 %) on March 9. Of all students, in 58 % (52 % intervention students and 64 % control students) there was information about all three measurement points. Multilevel analysis showed a statistically significant effect of time on the absence rate in periods (*F* = 196.0; *p* ≤ 0.01), and a significant interaction effect (*F* = 18.4; *p* ≤ 0.01) in favour of the intervention group. Figure 1 shows that in the intervention group the number of absence periods decreased over time: from 3.9 (95 % CI:3.8–4.1) to 2.5 (95 % CI:2.3–2.6) and finally to 2.2 (95 % CI:2.0–2.4) times reported sick in 12 school weeks. In the control group, the number of absence periods initially decreased from 4.5 (95 % CI:4.3–4.7) to 3.5 (95 % CI:3.3–3.6), after which an increase to 3.7 (95 % CI:3.5–3.9) was measured.

**Fig. 2:**
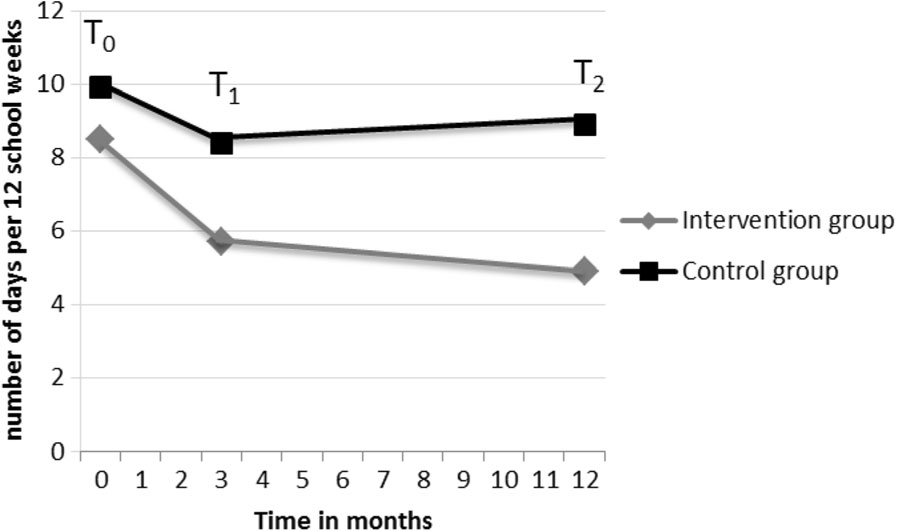
The progress of the absenteeism in number of days. Because of the application of a multilevel model for this part of the analysis, the initial value of 8.5 days differs from the initial value in Table 3

With respect to the absence rate in total number of days, there was a significant effect of time (*F* = 56.5; *p* ≤ 0.01) and a significant interaction effect (*F* = 11.5; *p* ≤ 0.01). Again the intervention group scored better than the control group. Figure 2 shows that in the intervention group the total number of absence days in the 12 week period decreased over time: from 8.5 (95 % CI: 7.9–9.1) to 5.7 (95 % CI: 5.0–6.2) to 4.9 (95 % CI: 4.2–5.6) days reported sick. In the control group, the number of absence days initially decreased from 9.9 (95 % CI: 9.3–10.5) to 8.4 (95 % CI: 7.9–9.0) days, after which an increase to 8.9 (95 % CI: 8.2–9.6) was measured.

**Fig. 3:**
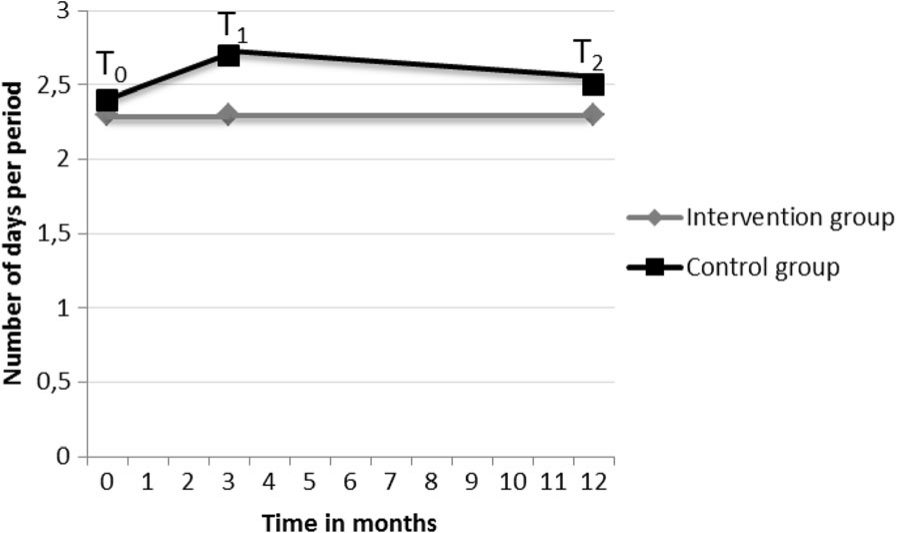
The progress of the absenteeism in number of days per period

With respect to the number of days per period, there was no significant effect of time (*F* = 1.0; *p* > 0.05) and no significant interaction effect (*F* = 1.3; *p* > 0.05). Figure 3 shows that in the intervention group the number of days per period remained the same over time: 2.3 (95 % CI: 2.1–2.6) at T_0_,2.3 (95 % CI: 2.1–2.7) at T_1_, and 2.3 (95 % CI: 1.9–2.6) at T_2_. In the control group, the number of days per period increased initially from 2.4 (95 % CI: 2.2–2.6) to 2.7 (95 % CI: 2.4–2.9) days, after which a decrease to 2.5 (95 % CI: 2.3–2.8) was measured.

A gender effect was found on the absence rate in periods (*F* = 8.1; *p* ≤ 0.01), indicating that, in general, girls had a somewhat higher absence rate (3.4) than boys (3.2). For the total number of days no statistically significant difference was found between boys and girls (*F* = 3.3; *p* > 0.05). For age no differences were found according to the absence rate in periods (*F* = 2.0; *p* > 0.05) and total number of days (*F* = 0.7; *p* > 0.05). In addition, no interaction effects were found of gender and time and of age and time on absence rate. The part of the variance that could be attributed to differences between schools was 7 % for the number of absence periods and 12 % for the number of absence days. The part of the variance that could be attributed to the students within schools was 23 % for the number of absence periods and 35 % for the number of absence days.

## Discussion

To the best of our knowledge, this was the first study of an intervention for addressing medical absenteeism in which schools and YHCPs collaborated intensively. The objective was to investigate the effects of MASS on students’ medical absenteeism in pre-vocational secondary schools, compared to a situation in which MASS was not applied.

The proportion of students having a high absent rate (four periods in 12 school weeks or more than six consecutive days) was 0.12 within the intervention schools versus a proportion of 0.14 within the control schools. This difference in proportion might be explained by an effect of the intervention at school level: increased awareness of and more attention to sickness reporting may within the school community result in a decrease in medical absenteeism. Moreover, under-identification of intervention students cannot be ruled out. The statistically significant difference between the two study groups related to gender (56 % of the intervention students and 64 % of the control students was male) suggests that schools may be less inclined to refer female students to the YHCP. A statistically significant difference in age between the two groups was also found. However, the actual difference was only 0.22 years (about 2½ months), which seems irrelevant in practice. Since there seemed to be no influence of gender and age on absence over time, probably this selection in the intervention group did not influence our results. The fact that there were more missing data in the intervention group can be explained by the different ways of inclusion between the control and intervention group. However, additional analyses showed no differences in the baseline measurement (as well in days as in periods) between the students with complete data and those with a missing on one of the measurement points.

Although the absence rate of all intervention and control students met one of the MASS-criteria, there were significant differences in the baseline measurements between both groups. This might be due to the way the data was obtained. In the intervention group, the baseline measurement was the consultation moment with the YHCP. The lower baseline absence rate in the intervention group might be explained by the attention and awareness generated by MASS during the time needed to actually meet the YHCP. In the control group, the data for the baseline measurement was close to the moment of meeting one of the criteria and was collected retrospectively. Meanwhile, in the control group there was no systematic attention, as in MASS, in case of an extensive absence rate. Consequently, it is conceivable that a part of the effect of MASS has already been measured at the baseline measurement and therefore cannot generate an effect anymore at follow-up measurements.

The study showed a decrease in the number of absence periods and absence days per 12 school weeks in both groups and in both measurements. The effects were significantly stronger in the intervention group. Regarding the initial decrease after 3 months in both groups, there are three possible interpretations. First, the decrease could (partly) be explained by “regression-to-the-mean” since only students with the highest rates of absenteeism were included in this study. However, the effect measured in the control group is less strong, therefore “regression-to-the-mean” cannot be sustained as the only explanation for the decline. Secondly, the influence of seasonality should be considered because (data of the) students were included at different times: 75.3 % of the intervention students were included before December 1, compared to 34.6 % of the control students. The influence of influenza is almost negligible because, in the Netherlands, in 2011–2012, the prevalence of influenza was extremely low [40]. Paediatric diseases, however, like gastroenteritis, functional complaints and asthma, must be taken into account as they have peaks from November till March [41]. Therefore, the first follow-up measurement in the control group has occurred more often in the peak season. This could explain the lesser decline at the first follow-up measurement in the control group. Thirdly, another reason for the rapid decline of absenteeism in the intervention group after 3 months may be due to the MASS intervention, which generated attention and care.

Regarding the effects after 12 months, it should be noticed that this follow-up measurement took place in the same period as the baseline measurement in both groups. There are two possible interpretations for the different outcomes in both groups. First, seasonality should be considered again. It is likely that seasonal influences of the pediatric diseases have had the same impact on both measurements. However, taking into account that there was a severe flu epidemic in the Netherlands, in 2012–2013, from January to March [40], this could have adversely affected the second follow-up measurement in the intervention group as the measurement was more often during the flu-peak. A difference between the second follow-up measurements can therefore partly be explained by the flu for the benefit of the control group. Secondly, the long-term effect after 12 months may be an effect of MASS. Regarding the number of days per period, no significant effects were found. Our findings are in line with a study of the 12-months effectiveness of an intervention called “SHARP-at work” for reducing recurrent sickness absence in workers with common mental disorders [42]. Arends et al. demonstrated that the intervention had especially an effect on the incidence of recurrent sickness absence.

It is likely that the results can be generalised to a national level. Although a statistically significant part of the variance could be attributed to the school level (7–12 %) the greatest effects were found at the individual student level (25–35 %). The effects that can be expected on other educational levels are uncertain and subject to future research. Strengths of the study are the large sample size and the intervention being rooted in Dutch health care and educational systems. It remains unclear to what extent these results can be generalised internationally, since both public health care and school systems differ substantially across countries.

Medical absence rate was chosen as the central outcome measurement in this study because the absence itself is related to a lower level of education and even school dropout. Consequently, this study provides no definite answers which factors are responsible for the decrease in absence rates that were found in this study. A combination of systematic, and thereby improved, identification of students with extensive medical absenteeism, the school paying more attention to students’ medical absenteeism, and referral to the YHCP, makes students feel that they and their absenteeism are not ignored but taken seriously. This can result in a raised threshold for reporting sick in future because students cannot avoid attention. The decrease in medical absenteeism can also be attributed to the intervention of the YHCP, resulting in more personalised and adequate treatment, care or support: after analysing the great variability of underlying reasons for the absenteeism [43], appropriate care, educational adjustments and adequate support could be arranged by schools and YHCPs in a sustainable manner. As a consequence, students possibly experience fewer (health-related) problems and/or find better ways to deal with their problems. Interventions are already available to handle with absence in cases of specific diseases and problems experienced by the student [2]. Our study showed a relatively high effect at the students’ level. For future research it is recommended to investigate these individual variations: to what extent could the differences in absence trajectories over time be explained by students’ characteristics such as coping behaviour, their social context such as family and peers, the underlying diseases and problems, and to the possible contributions of school, YHCP and health care characteristics. A multicentre prospective study in which intervention and control condition are located in different regions is recommended to further investigate the role of the school and their considerations in referring to the YHCP, to study the effects of the intervention on the care and support initiated by school and health care, and on the satisfaction of students and parents with care and support, and on students’ well-being and health.

## Conclusions

The study provides first indications of the added value of personalised management of medical absenteeism, i.e. systematic identification of students with extensive medical absenteeism and consistent referral to the YHCP, compared to “medical absenteeism policy as usual”. The effectiveness of the intervention is demonstrated primarily by a decrease in the number of periods reported sick. MASS seems to be a promising tool in the public health setting for addressing medical absenteeism among students.

## Appendix

Box 1 Description of the Dutch educational system.

**Table:**

	The Dutch general educational system is structured as follows (following the International Standard Classification of Education ISCED) [26]: at the age of 12, after primary education, students can choose between three tracks of education, each aiming at a different educational level in secondary school. They can go to pre-vocational secondary education (VMBO: ISCED 2), which is the lowest level, takes 4 years and prepares for intermediate vocational education (MBO: ISCED 3C). The second track in Dutch secondary education is senior general secondary education (HAVO), which takes 5 years and prepares for higher vocational education (HBO). The third track is pre-university education (VWO), which takes 6 years and prepares for studies at university level (academic bachelors and masters). Students can arrange their educational pathways in many ways, by going up or down levels. The levels relate to the degree of complexity of the content of the programme. In addition to general education, in the Netherlands there is also ‘special needs education’, in which the content of the educational programmes designed can be adapted to students’ specific needs.
